# UK veterinary professionals’ perceptions and experiences of adverse drug reaction reporting

**DOI:** 10.1002/vetr.1796

**Published:** 2022-06-03

**Authors:** Heather Davies, Gina Pinchbeck, Peter‐John M. Noble, Gillian Diesel, Munir Pirmohamed, Nadine Anderson, David R. Killick

**Affiliations:** ^1^ Institute of Infection, Veterinary and Ecological Sciences University of Liverpool Neston UK; ^2^ Pharmacovigilance Unit Veterinary Medicines Directorate Addlestone UK; ^3^ Wolfson Centre for Personalised Medicine, Institute of Systems, Molecular and Integrative Biology University of Liverpool Liverpool UK

## Abstract

**Background:**

Spontaneous reporting of suspected adverse drug reactions (ADRs) is the cornerstone of pharmacovigilance. Despite this, it is believed that there is significant under‐reporting in the veterinary setting. Low reporting rates delay marketing authorisation holders (MAHs) and regulators taking mitigating action in the case of safety concerns.

**Method:**

We designed a survey to explore the perceptions, attitudes and experiences of UK veterinary professionals towards ADR reporting. The survey was advertised widely through conventional and social media and at several conferences.

**Results:**

In total, 260 respondents completed the survey, including 210 veterinary surgeons, 49 veterinary nurses and one suitably qualified person. Respondents generally understood the need to report ADRs. The main barrier to reporting was the suspected ADR being well known, and the most popular potential facilitator identified was the ability to report via the practice management system. Facilitation via education in the form of a pharmacovigilance themed continuing professional development event was particularly popular among veterinary nurses, who reported time as being less of a barrier to reporting than their veterinary surgeon counterparts.

**Conclusions:**

Our findings suggest that technological interventions to facilitate reporting and empowerment of veterinary nurses to report through a tailored training event should be explored further.

## INTRODUCTION

Pharmacovigilance is defined by the World Health Organization as the science related to the detection, assessment, understanding and prevention of adverse events (AEs).^1^ An AE is any observation in animals, regardless of causal association, that is unfavourable and unintended occurring after the use of a veterinary medicinal product, including both on and off‐label usage.^2^ This includes lack of expected efficacy and events in humans following exposure to a veterinary product. If there is a reasonable possibility that the observed events were in response to an administered product, when the dose given was that normally used in animals for prophylaxis, diagnosis or therapy of disease, or modification of physiological function, then it may be possible to conclude that the AE is in fact an adverse drug reaction (ADR).^2^ However, in clinical practice, the distinction between these two terms is seldom considered and ‘ADR’ is frequently used synonymously to describe any AE.

The safety, quality and efficacy of marketed veterinary medicinal products are assessed before a marketing authorisation (MA) is granted.^3^ However, preapproval studies are typically short in duration and include only a small number of animals that are not as diverse as the target population.^4^ Moreover, once marketed, products are commonly used off‐label and/or under the prescribing cascade. Consequently, premarketing safety and efficacy studies cannot detect all AEs that may occur once a product is used in a wider population, highlighting the importance of postmarketing pharmacovigilance in the detection and mitigation of emerging ADRs.

Voluntary reporting of AEs by healthcare professionals and the public to national competent authorities and MA holders (MAHs), so‐called spontaneous reporting, is the most widely used methodology for collection of postmarketing safety data. In the United Kingdom (UK), the national competent authority for veterinary medicinal products is the Veterinary Medicines Directorate (VMD). There is no legal obligation to report suspected AEs in the UK; however, both veterinary surgeons and veterinary nurses are encouraged to report ‘suspected AEs’ by their respective Code of Professional Conduct.^5^, ^6^

While spontaneous reporting systems play an important role in the detection of AEs, there is evidence to suggest marked under‐reporting in both human and veterinary medicine. The under‐reporting rate in human medicine may be in excess of 90%,^7^ and the National Veterinary Medicines Agency in France estimated a similar under‐reporting rate.^8^ In a survey of veterinary practitioners across Europe, respondents indicated observing an average of one AE for every 100 treatments administered, but submitted an average of less than one report per year.^9^ An investigation of equine influenza vaccination in the UK estimated that respondents had observed 2760 ADRs in the last 12 months, of which 526 (19.1%, 95% CI: 17.6%–20.6%) had been reported.^10^ Similarly, surveys undertaken in Germany^11^ and Sweden^12^ also indicate under‐reporting by veterinary professionals.

The previous survey of ADR reporting in veterinary professionals across Europe attracted only a small number of responses from UK veterinary professionals.^9^ Therefore, this study set out to evaluate the barriers to reporting faced by veterinary professionals in the UK and identify opportunities to facilitate spontaneous reporting of AEs by exploring the perceptions, attitudes and experiences of veterinary professionals towards ADR reporting.

## METHODS

A cross‐sectional survey was developed using the JISC online survey tool. The survey consisted of five sections collecting data on respondent demographics, understanding of reporting requirements, personal experience of reporting, perceived barriers to reporting and perceived facilitators to reporting. Question styles included open and closed questions and five‐point Likert scales.

While the terms ‘adverse event’ and ‘adverse drug reaction’ have distinct regulatory meanings, they are used interchangeably by veterinary professionals, regulators (e.g., the Royal College of Veterinary Surgeons [RCVS]) and in significant parts of the pharmacovigilance literature. Therefore, in the context of this study, the terms ‘adverse drug reaction’ or ‘suspected adverse drug reaction’ were used for simplicity.

The project was approved by the University of Liverpool Veterinary Science Ethics Research Committee (reference number VREC789). The survey ran from 23 May 2019 to 26 January 2020, which included testing at the Small Animal Teaching Hospital at the University of Liverpool. The survey was accessed via a URL.

Extensive efforts were made to advertise the survey to veterinary professionals in the UK (veterinary professionals outside the UK were excluded) via the veterinary press (Vet Times, VetSurgeon.org website, Vet Nurse website and a letter in the Veterinary Record), social media groups (Vet Voices, Vet Nurse Wish List and Liverpool Veterinary Alumni Association) and in‐person at two veterinary conferences (London Vet Show and SPVS). Several organisations also distributed the survey to their members, including the University of Liverpool Veterinary Postgraduate Unit, BSAVA and BSAVA‐affiliated professional groups: Association of Veterinary Anaesthetists (AVA), British Veterinary Dermatology Study Group (BVDSG), Small Animal Medicine Society (SAMSoc) and Veterinary Cardiovascular Society (VCS). The BVA and RCVS Knowledge also promoted the study via their respective newsletters. Three corporate veterinary groups (CVS, Vets4Pets, Linnaeus) also distributed the survey directly to their practices. A prize draw was offered to encourage participation.

Descriptive statistics were produced for respondent demographics and for closed questions using Microsoft Excel and SPSS 25. Open questions were analysed by grouping similar themes. Univariable analysis was conducted using R v4.0.2 to explore the influence of five respondent characteristics on responses given to various questions: job role, area of practice, career stage, whether they had previously reported an ADR following the use of a veterinary medicinal product and type of practice. To investigate respondents' experiences of ADR reporting, *χ* ^2^ (or Fisher's exact tests) were used to explore differences between groups of veterinary professionals in relation to whether they had previously reported different types of ADRs. Mann–Whitney *U* tests were used to compare the number of reports submitted in the last 12 months for different groups of professionals.

## RESULTS

### Demographics

A total of 260 responses were available for analysis. Most respondents were veterinary surgeons (*n* = 210/260, 80.8%), while 18.8% (*n* = 49) of respondents were veterinary nurses (including one student veterinary nurse). The remaining respondent was a suitably qualified person (SQP).

The majority of respondents spent most or all of their time in small animal practice (91.8%, *n* = 236/257). However, 115 respondents indicated that they spent at least some of their time in exotic practice (44.2%, *n* = 115/260). In addition, a small proportion of respondents indicated spending at least some of their time in equine (10.4%, *n* = 27/259) or farm practice (6.5%, *n* = 17/260).

Two hundred respondents (76.9%) worked in first‐opinion practice. A small number of respondents (*n* = 6, 2.3%) indicated they worked in other settings, such as for a professional body, in a hospital setting, in a joint venture partnership or time split between two sectors.

The demographics of respondents are shown in Table 1.

**TABLE 1:** Demographics of 260 survey respondents showing profession, gender, age, years qualified and main sector of practice

		***n***	**%**
**Profession**	Veterinary surgeon	210	80.8%
	Veterinary nurse	49	18.8%
	Suitably qualified person (SQP)	1	0.4%
**Gender**	Female	195	75.0%
	Male	63	24.2%
	Prefer not to say	1	0.4%
	Prefer to self‐identify	1	0.4%
**Age (years)**	18–24	5	1.9%
	25–34	99	38.1%
	35–44	80	30.8%
	45–54	47	18.1%
	55–64	25	9.6%
	65+ years	4	1.5%
**Years qualified**	<1 year	3	1.2%
	1–5 years	52	20.0%
	6–10 years	58	22.3%
	11–15 years	41	15.8%
	16–25 years	55	21.2%
	>25 years	51	19.6%
**Sector of practice**	First‐opinion private practice	200	76.9%
	Referral practice (academic, private or other)	46	17.7%
	Charity practice	4	1.5%
	Industry	3	1.2%
	Non‐clinical (academic or other)	1	0.4%
	Other	6	2.3%

### Understanding of reporting requirements

**FIGURE 1:**
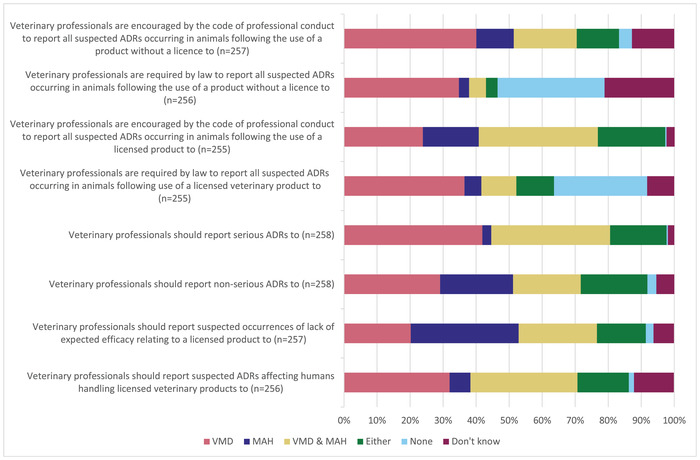
The organisation to which respondents believed they should submit an adverse drug reaction (ADR) report given various statements. MAH, marketing authorisation holder; VMD, veterinary medicines directorate

The majority of respondents believed that they should submit ADR reports following the use of both licensed and unlicensed products. However, 63.5% of respondents believed this was a legal requirement for licensed products (*n* = 162/255) and 46.5% (*n* = 119/256) believed that this was a legal requirement for unlicensed products. For serious ADRs, 36.0% (*n* = 93/258) of respondents believed that they should report to both the VMD and the MAH. Further details regarding the organisation to which respondents believed they should report ADRs are shown in Figure 1. Responses to this section for veterinary surgeons and veterinary nurses are shown in Table 2.

**TABLE 2:** The organisation to which respondents believe they should submit an adverse drug reaction (ADR) report given each statement, for veterinary surgeons (V) and veterinary nurses (VN)

		**VMD**	**MAH**	**VMD and MAH**	**Either**	**None**	**Don't know**
Veterinary professionals are required by law to report all suspected ADRs occurring in animals following the use of a licensed product to	V	36.9% (*n* = 76)	4.9% (*n* = 10)	6.8% (*n* = 14)	11.7% (*n* = 24)	31.1% (*n* = 64)	8.7% (*n* = 18)
	VN	34.7% (*n* = 17)	6.1% (*n* = 3)	26.5% (*n* = 13)	10.2% (*n* = 5)	16.3% (*n* = 8)	6.1% (*n* = 3)
Veterinary professionals are encouraged by the Code of Professional Conduct to report all suspected ADRs occurring in animals following the use of a licensed product to	V	23.2% (*n* = 48)	17.9% (*n* = 37)	34.3% (*n* = 71)	22.2% (*n* = 46)	0.0% (*n* = 0)	2.4% (*n* = 5)
	VN	27.1% (*n* = 13)	12.5% (*n* = 6)	43.8% (*n* = 21)	12.5% (*n* = 6)	2.1% (*n* = 1)	2.1% (*n* = 1)
Veterinary professionals are required by law to report all suspected ADRs occurring in animals following the use of a product without a licence to	V	33.8% (*n* = 70)	1.9% (*n* = 4)	2.4% (*n* = 5)	2.9% (*n* = 6)	36.2% (*n* = 75)	22.7% (*n* = 47)
	VN	38.8% (*n* = 19)	8.2% (*n* = 4)	16.3% (*n* = 8)	6.1% (*n* = 3)	16.3% (*n* = 8)	14.3% (*n* = 7)
Veterinary professionals are encouraged by the Code of Professional Conduct to report all suspected ADRs occurring in animals following the use of a product without a licence to	V	40.9% (*n* = 85)	10.6% (*n* = 22)	18.3% (*n* = 38)	13.5% (*n* = 28)	3.8% (*n* = 8)	13.0% (*n* = 27)
	VN	36.7% (*n* = 18)	14.3% (*n* = 7)	22.4% (*n* = 11)	10.2% (*n* = 5)	4.1% (*n* = 2)	12.2% (*n* = 6)
Veterinary professionals should report serious ADRs to	V	43.5% (*n* = 91)	1.9% (*n* = 4)	33.0% (*n* = 69)	19.1% (*n* = 40)	0.5% (*n* = 1)	1.9% (*n* = 4)
	VN	34.7% (*n* = 17)	6.1% (*n* = 3)	49.0% (*n* = 24)	8.2% (*n* = 4)	0.0% (*n* = 0)	2.0% (*n* = 1)
Veterinary professionals should report non‐serious ADRs to	V	30.1% (*n* = 63)	20.6% (*n* = 43)	20.6% (*n* = 43)	20.6% (*n* = 43)	3.3% (*n* = 7)	4.8% (*n* = 10)
	VN	24.5% (*n* = 12)	28.6% (*n* = 14)	20.4% (*n* = 10)	18.4% (*n* = 9)	0.0% (*n* = 0)	8.2% (*n* = 4)
Veterinary professionals should report suspected occurrences of lack of expected efficacy relating to a licensed product to	V	21.2% (*n* = 44)	30.8% (*n* = 64)	24.0% (*n* = 50)	15.9% (*n* = 33)	1.9% (*n* = 4)	6.3% (*n* = 13)
	VN	16.3% (*n* = 8)	40.8% (*n* = 20)	22.4% (*n* = 11)	10.2% (*n* = 5)	4.1% (*n* = 2)	6.1% (*n* = 3)
Veterinary professionals should report suspected ADRs affecting humans handling licensed veterinary products to	V	30.9% (*n* = 64)	5.8% (*n* = 12)	31.4% (*n* = 65)	16.9% (*n* = 35)	1.4% (*n* = 3)	13.5% (*n* = 28)
	VN	36.7% (*n* = 18)	8.2% (*n* = 4)	36.7% (*n* = 18)	10.2% (*n* = 5)	2.0% (*n* = 1)	6.1% (*n* = 3)

Abbreviations: MAH, marketing authorisation holder; VMD, veterinary medicines directorate.

With regards to MAH responsibilities, a total of 82.7% (*n* = 196/237) of respondents correctly believed that MAHs are required by law to submit all serious ADR reports that they receive to the VMD. Furthermore, 64.1% (*n* = 166/259) believed that MAHs are required by law to submit all ADR reports to the VMD.

### Experience of ADR reporting

While most respondents had previously reported an ADR following the use of a licensed product (83.5%, *n* = 197/236), only 9.7% (*n* = 23/236) of respondents had reported an ADR following the use of an unlicensed product. Over a quarter of respondents had previously reported a suspected lack of expected efficacy (SLEE) event (28.2%, *n* = 73/259). Analysis revealed that veterinary surgeons were more likely than veterinary nurses to have reported an ADR in relation to a licensed product and a SLEE event (*p* < 0.001 and 0.04, respectively).

In the 12 months preceding the survey, respondents (*n* = 257) indicated that they had reported between 0 and 20 ADRs. The total number of ADRs reported was 487, and the median was one. Veterinary surgeons reported submitting ADR reports more frequently than veterinary nurses (*p* < 0.001) and first‐opinion practitioners reported submitting reports more frequently than those working in referral practice (*p* < 0.001) (Table 3).

**TABLE 3:** Number of adverse drug reactions (ADRs) submitted in the last 12 months by various groups of veterinary professionals

	**Number of ADR reports submitted in last 12 months**					
		**Total**	**Median**	**Interquartile range**	**Range**	***p*‐Value**
Profession	Veterinary surgeon (*n* = 208)	455	1	3	0–20	<0.001
	Veterinary nurse (*n* = 48)	32	0	1	0–6	
Area of practice	Large animal practice (*n* = 29)	41	1	2	0–10	0.18
	Small animal practice (*n* = 177)	414	1	3	0–20	
Stage of career	Veterinary surgeons					
	≤10 years qualified (*n* = 89)	201	1	2	0–20	0.81
	>10 years qualified (*n* = 119)	254	1	3	0–20	
	Veterinary nurses					
	≤5 years qualified (*n* = 15)	9	0	1	0–3	0.68
	>5 years qualified (*n* = 33)	23	0	0	0–6	
Type of practice	First‐opinion practice (*n* = 164)	428	2	2	0–20	<0.001
	Referral practice (*n* = 34)	18	0	1	0–4	

Respondents (*n* = 257) indicated that, in the last 12 months, they had reported a total of 84 SLEE events, ranging from 0 to 10 with a median of 0. Overall, 40.9% (*n* = 106/259) of respondents indicated that they had never observed a SLEE event. Of the respondents who had observed SLEE events, 74.5% (*n* = 114/153) indicated that these were most frequently observed in relation to licensed products, compared to 25.5% (*n* = 39) respondents who indicated that they most frequently observed SLEE events following the use of an unlicensed product.

To explore the actions taken by veterinary professionals when an ADR is suspected, respondents were asked to think of the last such event they had observed. Most respondents had previously observed an ADR in relation to a licensed product (95.0%, *n* = 224/236), whereas suspected ADRs after the use of unlicensed products were less frequently observed (39.8%, *n* = 94/236). ADRs after licensed products appear more likely to be reported (85.3%, *n* = 191/224) than for unlicensed (23.4%, *n* = 22/94). Table 4 shows the action taken by respondents following observation of an ADR in relation to licensed and unlicensed products.

**TABLE 4:** Actions taken by respondents in relation to the last adverse drug reaction (ADR) they observed following the use of licensed and unlicensed products

**Thinking about the last suspected ADR that you observed—what did you do?**				
	**Licensed products (*n* = 236)**		**Unlicensed products (*n* = 236)**	
	***N***	**%**	***n***	**%**
Never observed an ADR	12	5.1	142	60.2
ADR reported	63	26.7	9	3.8
ADR reported and recorded in consultation notes	128	54.2	13	5.5
ADR recorded in consultation notes	28	11.9	49	20.7
No action	3	1.3	23	9.8
Other	6^a^	2.5	2^b^	0.9

^a^ Four respondents also selected other actions as part of their response.

^b^ Both respondents also selected other actions as part of their response.

Free‐text responses were used to expand on the action taken when a respondent had selected ‘Other’. For licensed products, these responses were recording the ADR in the clinical governance record (*n* = 1) and advising another member of staff (*n* = 3) (i.e., reporting to head of clinical governance, requesting a daytime member of staff report the ADR or advising colleagues involved in dispensing). One respondent indicated that they had suggested reporting the event but were told not to, and a further respondent could not recall the action they had taken. For unlicensed products, respondents indicated that they had advised someone else about the ADR, either daytime practice staff (*n* = 1) or warning colleagues to take care with the product as well as informing owners what to look out for (*n* = 1).

As shown in Table 4, a small number of respondents indicated that they took no action following observation of an ADR. For licensed products, explanations for inaction included not having the time (66.6%, *n* = 2/3), the ADR being non‐serious (100.0%, *n* = 3/3), there being no legal obligation to report (33.3%, *n* = 1/3) and not knowing how to report (33.3%, *n* = 1/3). For unlicensed products, 13.0% (*n* = 3/23) of respondents indicated a lack of time, the ADR being known and/or non‐serious were also reasons for taking no action (8.7%, *n* = 2/23 and 34.7%, *n* = 8/23, respectively). Other explanations included not knowing how to report (26.1%, *n* = 6/23) and there being no legal obligation to report (39.1%, *n* = 9/23). Four respondents indicated ‘other’ and free‐text explanations offered indicated that respondents were not aware that they should report ADRs due to an unlicensed product.

**FIGURE 2:**
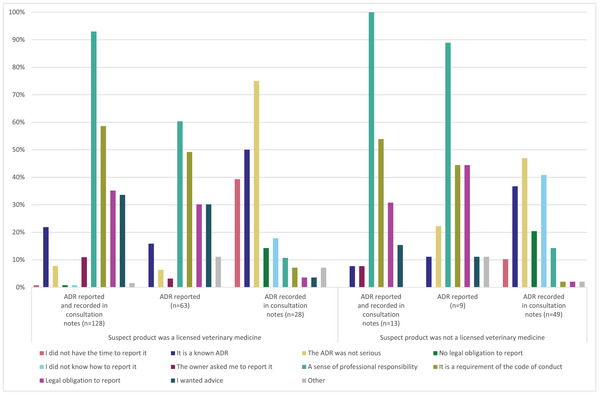
Explanations given to support respondents’ action taken following observation of adverse drug reaction (ADR) in relation to licensed and unlicensed products

A sense of professional responsibility and it being a requirement of the Code of Professional Conduct appeared to be the main drivers for reporting ADRs for both licensed and unlicensed products. The type of event (i.e., non‐serious or known ADR) appeared to influence the decision to record the ADR in consultation notes only for both types of products. Not knowing how to report an ADR following the use of an unlicensed product was an important factor in choosing to record the ADR in the consultation notes only. Figure 2 shows the three types of action taken for ADRs observed following licensed and unlicensed products and the explanations given by respondents for taking those actions.

### Barriers to reporting

**FIGURE 3:**
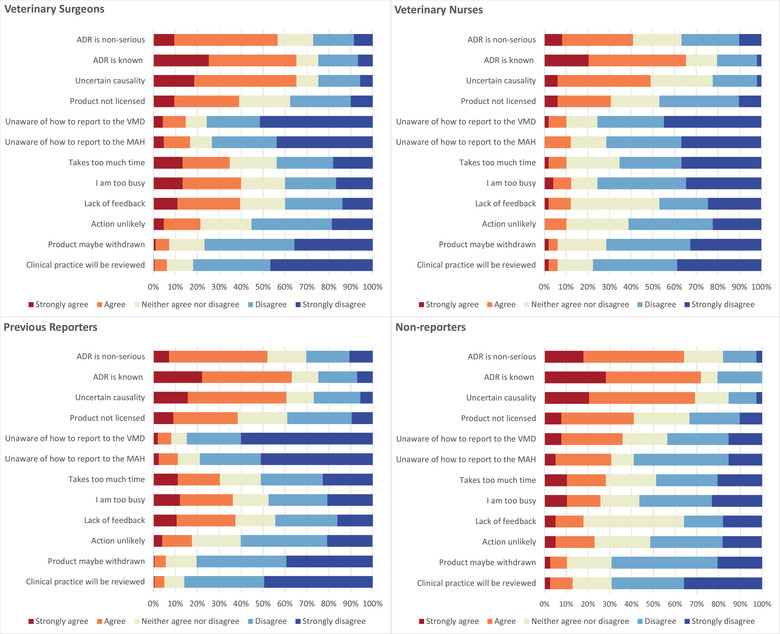
Level of agreement as indicated by different groups of veterinary professionals that the given factors would influence the decision to not report an adverse drug reaction (ADR). Factors in full: (i) the suspected ADR is non‐serious; (ii) the suspected ADR is already reported within the product literature (i.e., is a known side effect); (iii) uncertain that the ADR was caused by the product; (iv) the product was not licensed for use in the animal or indication concerned; (v) unaware of how to report to the Veterinary Medicines Directorate (VMD); (vi) unaware of how to report to the marketing authorisation holder (MAH); (vii) the reporting process takes too much time; (viii) I am too busy; (ix) lack of feedback from reports made; (x) feel that the VMD or MAH are unlikely to act on the report; (xi) concern that an effective product may be withdrawn; and (xii) concern that my clinical practice will be reviewed

When asked more generally about the barriers to reporting, respondents indicated that the three biggest barriers were the ADR being known (i.e., already in the product literature) (65.0% agreement, *n* = 169/260), the ADR being non‐serious (53.4% agreement, *n* = 139) and uncertainty whether the reaction was caused by the product (61.9% agreement, *n* = 161). Concern about product withdrawal and concern that clinical practice would be reviewed were rarely perceived as major barriers to reporting (Figure 3).

These three main barriers were the same for both veterinary surgeons and veterinary nurses; however, time constraints appeared to be a larger barrier for veterinary surgeons. Some 12.3% (*n* = 6/49) of nurses indicated that they agree or strongly agree that being too busy influences their decision to not report an ADR, whereas 40.0% (*n* = 84/210) of veterinary surgeons agreed with this statement. Similarly, 10.2% (*n* = 5) of veterinary nurses agreed (or strongly agreed) that the reporting process taking too much time influences their decision, with 34.7% (*n* = 73) of veterinary surgeons indicating the same. Over a third of veterinary surgeons (39.6%, *n* = 83) agreed that lack of feedback from reports made would influence the decision to not report an ADR, whereas only 12.2% (*n* = 6) of veterinary nurses agreed with this statement.

Among respondents who had not previously submitted a report, a lack of awareness of how to report to the VMD and MAH was perceived as a barrier to reporting by 35.9% (*n* = 14/39) and 30.7% (*n* = 12), respectively. Over a third of respondents (37.5%, *n* = 74/198) who had previously submitted an ADR report indicated that they agreed (or strongly agreed) that lack of feedback from submitted reports would influence their decision to not submit a report, whereas just 17.9% (*n* = 7) of respondents who had not previously submitted an ADR report indicated the same.

Figure 3 shows the full list of barriers given to respondents and the level of agreement indicated by veterinary surgeons, veterinary nurses, respondents who had previously submitted an ADR report and respondents who had not previously submitted a report, for each given statement.

### Facilitators of reporting

When respondents were asked about various facilitators to reporting, there was a good level of support for all the proposed options. Overall, the ability to report suspected ADRs via the practice management system (PMS) (81.9%, *n* = 213/260), follow‐up regarding action taken in relation to a report (76.9%, *n* = 200) and the ability to view reported ADRs on a dashboard within the PMS (71.6%, *n* = 186) were the most popular proposed facilitators.

Follow‐up regarding what action was taken by the VMD or MAH was a more popular choice among non‐reporters, with 87.1% (*n* = 34/39) of respondents indicating that this would make it more likely or a lot more likely that they would submit an ADR report, whereas 73.2% (*n* = 145/198) of previous reporters indicated the same.

Veterinary nurses (79.6%, *n* = 39/49) were more supportive of a CPD event regarding ADR reporting than veterinary surgeons (47.6%, *n* = 100/210). Similarly, respondents who had not previously submitted an ADR report were more in favour of a CPD event (*n* = 32/39, 82.0%) than those who had previously reported (*n* = 93/198, 46.9%).

**FIGURE 4:**
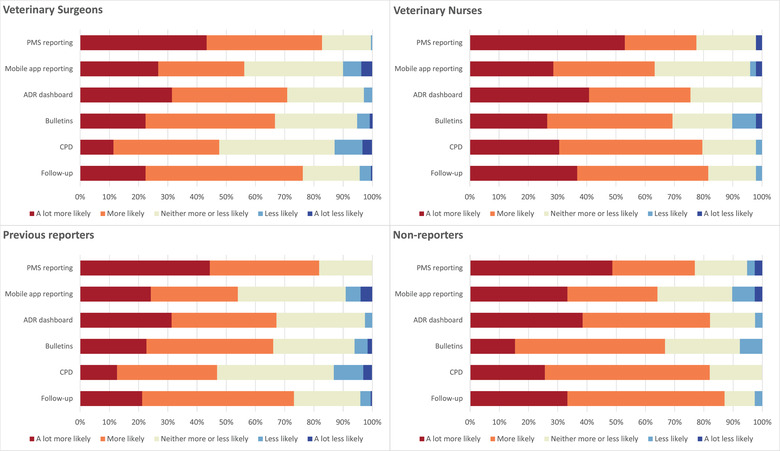
Likelihood that the given factors would make it more or less likely that respondents would report a suspected adverse drug reaction (ADR) or occurrence of lack of expected efficacy as indicated by veterinary surgeons, veterinary nurses, respondents who had previously reported an ADR and respondents who had not previously reported an ADR. Factors in full: (i) the ability to report suspected ADRs or occurrences of lack of expected efficacy through the practice management system (PMS); (ii) the ability to report suspected ADRs or occurrences of lack of efficacy through a mobile app; (iii) the ability to view suspected ADRs on a dashboard in the PMS; (iv) bulletins reporting the monitoring of licensed and unlicensed products in animals in the veterinary press, for example Vet Record or Vet Times; (v) CPD event regarding reporting ADRs in veterinary practice; (vi) follow‐up regarding what action was taken by the Veterinary Medicines Directorate and/or marketing authorisation holder

Figure 4 shows each proposed facilitator and the likelihood that each would make it more or less likely that respondents would submit an ADR report as indicated by veterinary surgeons, veterinary nurses, respondents who had previously submitted an ADR report and respondents who had not previously submitted a report.

## DISCUSSION

Effective postapproval pharmacovigilance is reliant on the voluntary reporting of suspected ADRs by veterinary professionals and members of the public. Despite this, there is growing evidence for under‐reporting within the veterinary setting.^8^, ^9^, ^10^, ^11^ Under‐reporting will contribute to delays in identifying emerging safety issues and lead to inaccurate estimates of ADR incidence rates (both broadly and in relation to specific drug‐event combinations). This study explored UK veterinary professionals’ understanding of their pharmacovigilance responsibilities, experiences of reporting and perceived barriers and facilitators.

The results show that the respondents understand their responsibility to report suspected ADRs. There is some confusion about the basis of this responsibility; while most respondents correctly understood the guidance in the RCVS Code of Professional Conduct, many also incorrectly believed there was a legal requirement to report. This finding is interesting in the context of countries that have moved to make ADR reporting a legal requirement for veterinary professionals as it could be interpreted in two ways. One way to interpret the results is that they suggest such moves may not dramatically alter reporting behaviour, as shown elsewhere.^13^ A second is that the respondents are biased towards a view that reporting is a legal requirement and because of this (erroneous) belief, they are more engaged with their professional responsibilities in this area than the profession in general. As more countries are moving towards mandating reporting, it will be interesting to see whether this influences reporting rates.

There seemed to be some uncertainty regarding which organisation different types of ADRs (i.e., serious, non‐serious or human reactions) and SLEE events should be reported to. Similar to De Briyne et al.,^9^ the three main barriers to reporting were the ADR being known (already in the product literature), uncertainty about whether the ADR was caused by the product and the ADR being non‐serious. These factors have also been identified as barriers to ADR reporting in human healthcare.^14^, ^15^, ^16^ In the veterinary setting, current guidance is that reporters can choose whether to report to the VMD or to the MAH and that professionals are encouraged to submit a report in all three of these instances. Improving pharmacovigilance education to include a description of the statistical processes employed to account for false positives may help to improve reporting rates where causality is not clear. In addition to this, highlighting the role that reporting non‐serious and known events plays in determining accurate product safety profiles may help to overcome these barriers to reporting. A simplified method for reporting common AEs may also help to increase reporting of these events, which in turn could increase confidence in the AE frequencies reported in the product information.

It is likely that there is a difference in how event seriousness is perceived by pet owners, clinicians and regulators. Interestingly, a recent change in EU guidance means that serious and non‐serious reports are processed in the same manner. In the UK, the Veterinary Medicines Regulations are currently being reviewed. This presents an opportunity to consider whether such a move would also be beneficial in the UK by removing confusion for reporters to make it so that all AEs are handled in the same way.

This survey also suggests that some reporters chose to report both to the VMD and MAH, and that veterinary nurses are more likely to do this. While there are processes in place to identify duplicates, it should be noted that this represents a duplication in effort on the part of the veterinary professional. It is recognised that many veterinary professionals will seek advice from the MAH and not realise that the MAH will in turn report this as an AE to the VMD. In this instance, MAHs are best placed to confirm to veterinary professionals when a report will be submitted to the VMD to make the process more efficient. Improved signposting both on the VMD webpage and by MAHs might reduce this duplication.

A thread running through the answers of non‐reporters is a lack of knowledge of the functioning of the pharmacovigilance system and a desire to know more. A standardised pharmacovigilance curriculum provided to all veterinary professionals may help enhance knowledge among veterinary professionals. Provision of pharmacovigilance education is the most frequent form of intervention used to increase reporting rates in the published literature; however, it has been found that studies consisting of multiple interventions typically result in greater increases in reporting than single interventions alone.^17^, ^18^

Similar to previous surveys of veterinary professionals, we conclude that respondents value feedback,^9^, ^12^ given that lack of feedback was revealed to be a significant barrier to reporting. In keeping with this, information about how a report was utilised by the MAH and VMD was noted to be a significant facilitator of reporting. Feedback might come in either an individualised or aggregate form.

Regarding aggregate feedback, the VMD and European Medicines Agency (EMA) both publish annual pharmacovigilance reports providing information on AEs under review and any mitigating action taken.^19^, ^20^ Additionally, the EMA provides some summary statistics related to suspected ADRs reported via a dedicated website (https://www.adrreports.eu), and the VMD publishes a monthly medicines update, which includes changes made to product information. This finding could therefore indicate a lack of awareness of these reports or dissatisfaction with their content. In fact, a previous study has suggested that up to 81% of veterinary professionals are unaware of the EMA public bulletin.^12^ Given this, increasing awareness of such bulletins seems pertinent. While VMD advertises their bulletin in the Vet Record, it should be acknowledged that this publication is only free to BVA members. Adding additional lines of communication in a freely available context such as social media may improve awareness. Moreover, regulators could seek to work with professional bodies such as the RCVS and via quality improvement leads within corporate veterinary groups to enhance distribution. In addition to improving delivery, consideration could also be given to format. It seems logical that veterinary professionals may engage with information more effectively if it is presented in an accessible and clinically relevant format. For example, the data summaries such as those on the EMA website could include more information that is useful to clinicians in practice; for example, descriptions of events and their outcome (reported at a more granular level than system organ class). Clearly, regulators would need to consider the balance of possible benefits of a more open approach against the risk of releasing information about drug‐event combinations before the relationship is proven to be statistically robust and the risk of releasing commercially sensitive information if a particular product is identifiable.

Individualised case feedback is problematic due to both the large volume of reports submitted and because uncertainty around individual reports means that statistical approaches are necessary to identify safety signals among considerable statistical noise. As it is rarely possible to be certain that an individual report truly pertains to an ADR, there is risk that individualised feedback may give a false view of the current knowledge base and risk. Nonetheless, feedback has also been shown to influence the willingness to report in human healthcare.^21^ Beside individualised case feedback, it may be that providing feedback on how the data feeds into the pharmacovigilance system might enhance understanding of the value of reporting. Therefore, further studies to understand whether and what type of feedback might encourage reporting by veterinary professionals are required.

This study highlighted the role of veterinary nurses in the pharmacovigilance system. Veterinary nurses currently report fewer ADRs than veterinary surgeons, but were more receptive to a CPD event as a facilitator and were less concerned by the lack of feedback on submitted reports. This demonstrates the willingness of many veterinary nurses to engage in the current pharmacovigilance framework. Moreover, we found that the time taken to report, and being too busy to report, was less of a barrier for veterinary nurses, suggesting that veterinary nurses may have more capacity to support pharmacovigilance processes. Given this, it would be interesting to evaluate whether veterinary nurses could undertake a specific role in ADR reporting.

Appointing a responsible person for ADR reporting has been shown to be an effective strategy for increasing report submission frequency.^22^ Similarly, appointing ‘champions’ to provide education and training on pharmacovigilance effectively increased reporting at human hospitals in the UK.^23^ Nurses are increasingly involved in ADR reporting in human healthcare,^24^, ^25^, ^26^ and the quality of reports submitted by nurses is comparable to those of doctors.^27^ A recent survey of veterinary professionals in Sweden indicated that veterinary nurses are aware of significant numbers of ADRs that have gone unreported by veterinary surgeons in their respective practices,^12^ suggesting that veterinary nurses are well positioned to identify ADRs and report them if they are empowered to do so through education and training.

A practical suggestion would be to incorporate a requirement to have a named veterinary nurse responsible for AE reporting as part of the RCVS practice standards scheme or the revision of the Veterinary Medicine Regulations. Appointed veterinary nurses could be provided with specific training regarding AE reporting and be supported to discuss reporting and to collect details of AEs with colleagues during clinical meetings.

This study demonstrated that time is a factor for many veterinary professionals. It is therefore understandable that a voluntary, unpaid task is often overlooked. There is limited evidence to suggest that financial incentives^28^, ^29^ could help to increase the reporting rate; however, consideration should be given to who would be expected to bear this cost.

The ability to report ADRs via PMS was perceived as a potential facilitator to reporting; this seems consistent with responses suggesting lack of time as a barrier to reporting. Electronic reporting linked to electronic health records is a developing area of interest and was successfully piloted in general practice (human healthcare) in the UK.^30^ The pilot demonstrated a 43.8% increase in reporting from general practitioners, and consequently this technology is being rolled out to general practices across the UK. Methods such as embedding hyperlinks to existing online report forms in electronic health records and onto computer desktops have been shown to be effective in increasing reporting rates in Portugal,^31^ and reporting via the PMS was the preferred choice of reporting methods among veterinary surgeons in Sweden.^12^ This approach was also piloted in community pharmacy in Australia and was found to be a successful method for increasing the number of reports received.^32^ Interestingly, reporting via the PMS was not a high priority for veterinary professionals in Germany,^11^ demonstrating the importance of researching perceived facilitators at a national level.

Historically, electronic reporting via the PMS has not been available in the veterinary setting in the UK. Based on the results of this study, we have developed and launched an ADR reporting button for practices participating in the Small Animal Veterinary Surveillance Network (SAVSNET) project.^33^ The ADR reporting button is available to clinicians at the end of every consultation and provides functionalities such as prepopulation of animal and drug information and the option to append clinical notes to the report. Reports are submitted directly to the VMD via SAVSNET.

For this work, we chose to focus on two respondent variables: (a) job role (veterinary surgeon or veterinary nurse); and (b) previous reporting behaviour level (previous reporter or non‐reporter), as they provide the most direct route to interventions that could be targeted at individual groups to encourage reporting. It is of course feasible that a larger study that supported multivariable modelling might have yielded additional information.

The main limitation of this study is that it is a self‐selecting sample of veterinary professionals and relied on respondents being aware of the study and choosing to take part. Respondents to this survey represent less than 1% of all registered veterinary professionals in the UK; however, if their indicated reporting behaviour was typical of UK veterinary professionals, a much larger number of reports would be received by the VMD and MAHs. Whether this represents recall bias regarding reporting or a population of respondents with a greater than average interest in drug safety is uncertain. Furthermore, only a small proportion of respondents to this study indicated that they spent time in farm animal practice. While this is in line with the overall picture in the UK, it should be acknowledged that working practices are likely to be different for this group of professionals, meaning that further research into the specific barriers faced by those working in farm animal practice may provide insights into unique facilitators applicable to this group.

Future research should also consider alternative sources of pharmacovigilance data. One area of increasing interest is the use of electronic health records to supplement spontaneous reporting systems.^34^, ^35^ Electronic health records have many advantages for pharmacovigilance, not least as this does not rely on the active submission of a report.

## CONCLUSION

This study explored the understanding, experiences, barriers and facilitators to ADR reporting of UK veterinary professionals. The results indicated a good understanding of the requirement to report ADRs, largely driven by the RCVS Code of Professional Conduct. However, it highlights differences in understanding between veterinary surgeons and veterinary nurses. Educational interventions and empowerment of specific veterinary professional groups, coupled with technological innovations such as reporting via the PMS might improve reporting rates and are worthy of further investigation.

## CONFLICT OF INTEREST

The authors have no conflicts of interest to declare.

## AUTHOR CONTRIBUTIONS

David R. Killick and P.‐J. M. Noble conceived the project. Heather Davies, Gina Pinchbeck, P.‐J. M. Noble, Gillian Diesel, Munir Pirmohamed, Nadine Anderson and David R. Killick were involved in designing and distributing the survey. Statistics and analyses were performed by Heather Davies. The first draft of the manuscript was written by Heather Davies with significant editing, revision and contributions from Gina Pinchbeck and David R. Killick. All authors were involved in the writing of the final manuscript.

## Data Availability

The data that support the findings of this study, including a copy of the survey, are available from the corresponding author upon reasonable request.

## References

[1] World Health Organisation. Pharmacovigilance: ensuring the safe use of medicines. World Health Organisation; 2004. Accessed April 19, 2019. https://apps.who.int/iris/handle/10665/68782

[2] VICH. VICH GL24 on pharmacovigilance of veterinary medicinal products: management of adverse event reports (AERs). VICH; 2007. Accessed August 24, 2021. https://www.ema.europa.eu/en/documents/scientific‐guideline/vich‐gl24‐guideline‐pharmacovigilance‐veterinary‐medicinal‐products‐management‐adverse‐event‐reports_en.pdf

[3] Woodward KN . Veterinary pharmacovigilance. Part 1. The legal basis in the European Union. J Vet Pharmacol Ther. 2005;28(2):131–47. 15842304 10.1111/j.1365-2885.2005.00645.x

[4] O'Rourke D . The practitioner's role in SAR reporting. In Pract. 2008;30(7):398–402.

[5] Code of Professional Conduct for Veterinary Surgeons. Royal College of Veterinary Surgeons; 2018. Accessed March 10, 2019. https://www.rcvs.org.uk/setting‐standards/advice‐and‐guidance/code‐of‐professional‐conduct‐for‐veterinary‐surgeons/

[6] Code of Professional Conduct for Veterinary Nurses. Royal College of Veterinary Surgeons; 2018. Accessed March 10, 2019. https://www.rcvs.org.uk/setting‐standards/advice‐and‐guidance/code‐of‐professional‐conduct‐for‐veterinary‐nurses/

[7] Hazell L , Shakir SAW . Under‐reporting of adverse drug reactions : a systematic review. Drug Saf. 2006;29(5):385–96. 16689555 10.2165/00002018-200629050-00003

[8] Fresnay E , Laurentie S , Orand J . Under‐reporting in veterinary pharmacovigilance: study of adverse effects due to veterinary medicinal products. Bulletin des GTV; 2015. p. 95–102. Accessed August 24, 2021. https://www.anses.fr/fr/system/files/Resume%20EN%20pour%20site%20Anses‐Final.pdf

[9] De Briyne N , Gopal R , Diesel G , Iatridou D , O'Rourke D . Veterinary pharmacovigilance in Europe: a survey of veterinary practitioners. Vet Rec Open. 2017;4(1):e000224. 28848652 10.1136/vetreco-2017-000224 PMC5554794

[10] Wilson A , Pinchbeck G , Dean R , McGowan C . Equine influenza vaccination in the UK: current practices may leave horses with suboptimal immunity. Equine Vet J. 2021;53(5):1004–14. 33124070 10.1111/evj.13377 PMC8451788

[11] Sander S , Böhme B , McDaniel C . How can we make reporting ADRs easier? BVL survey at the Leipzig Veterinary Congress. Dtsch Tierärzteblatt. 2020;(68):1113–6.

[12] Mount J , Sjöström K , Arthurson V , Kreuger S . A survey of veterinary professionals in Sweden: Adverse event reporting and access to product safety information. Vet Rec Open. 2021;8(1):e18. 34386242 10.1002/vro2.18 PMC8342559

[13] Karlsson SA , Jacobsson I , Boman MD , Hakkarainen KM , Lövborg H , Hägg S , et al. The impact of a changed legislation on reporting of adverse drug reactions in Sweden, with focus on nurses’ reporting. Eur J Clin Pharmacol. 2015;71(5):631–6. 25845655 10.1007/s00228-015-1839-6

[14] Belton KJ , Lewis SC , Payne S , Rawlins MD , Wood SM . Attitudinal survey of adverse drug reaction reporting by medical practitioners in the United Kingdom. Br J Clin Pharmacol. 1995;39(3):223–6. 7619660 10.1111/j.1365-2125.1995.tb04440.x PMC1364995

[15] Hughes ML , Weiss M . Adverse drug reaction reporting by community pharmacists—the barriers and facilitators. Pharmacoepidemiol Drug Saf. 2019;28(12):1552–9. 31131966 10.1002/pds.4800

[16] Cheema E , Haseeb A , Khan TM , Sutcliffe P , Singer DR . Barriers to reporting of adverse drugs reactions: a cross sectional study among community pharmacists in United Kingdom. Pharm Pract. 2017;15(3):931–8. 10.18549/PharmPract.2017.03.931 PMC5597805 28943977

[17] Li R , Zaidi STR , Chen T , Castelino R . Effectiveness of interventions to improve adverse drug reaction reporting by healthcare professionals over the last decade: a systematic review. Pharmacoepidemiol Drug Saf. 2020;29:1–8. 10.1002/pds.4906 31724270

[18] Gonzalez‐Gonzalez C , Lopez‐Gonzalez E , Herdeiro MT , Figueiras A . Strategies to improve adverse drug reaction reporting: a critical and systematic review. Drug Saf. 2013;36(5):317–28. 23640659 10.1007/s40264-013-0058-2

[19] European Medicines Agency. Veterinary pharmacovigilance 2019 annual bulletin. European Medicines Agency; 2020. Accessed November 22, 2021. https://www.ema.europa.eu/en/documents/newsletter/public‐bulletin‐veterinary‐pharmacovigilance‐2019_en.pdf

[20] Veterinary Medicines Directorate. Veterinary pharmacovigilance in the UK annual review 2019 – a summary of veterinary adverse events. Veterinary Medicines Directorate; 2020. Accessed November 22, 2021. https://www.gov.uk/government/publications/veterinary‐medicines‐pharmacovigilance‐annual‐review‐2019‐summary

[21] Wallerstedt SM , Brunlöf G , Johansson ML , Tukukino C , Ny L . Reporting of adverse drug reactions may be influenced by feedback to the reporting doctor. Eur J Clin Pharmacol. 2007;63(5):505–8. 17347804 10.1007/s00228-007-0270-z

[22] Lander AR , Blicher TM , Jimenez‐Solem E , Jespersen M , Kampmann JP , Christensen HR . Introducing an adverse drug event manager. Eur J Hosp Pharm. 2013;20(2):78–81.

[23] Annual report 2013–2014. Yellow Card Centre Wales; 2014. Accessed August 24, 2021. https://openrepository.awttc.uk/app/serve/resource/mdbr0346

[24] Ulfvarson J , Mejyr S , Bergman U . Nurses are increasingly involved in pharmacovigilance in Sweden. Pharmacoepidemiol Drug Saf. 2007;16(5):532–7. 17072915 10.1002/pds.1336

[25] Conforti A , Opri S , D'Incau P , Sottosanti L , Moretti U , Ferrazin F , et al. Adverse drug reaction reporting by nurses: analysis of Italian pharmacovigilance database. Pharmacoepidemiol Drug Saf. 2012;21(6):597–602. 22337264 10.1002/pds.3225

[26] Sri Ranganathan S , Houghton JE , Davies DP , Routledge PA . The involvement of nurses in reporting suspected adverse drug reactions: Experience with the meningococcal vaccination scheme. Br J Clin Pharmacol. 2003;56(6):658–63. 14616426 10.1046/j.1365-2125.2003.01903.x PMC1884300

[27] Morrison‐Griffiths S , Walley TJ , Park BK , Breckenridge AM , Pirmohamed M . Reporting of adverse drug reactions by nurses. Lancet. 2003;361:1347–8. 12711472 10.1016/S0140-6736(03)13043-7

[28] Bäckström M , Mjörndal T . A small economic inducement to stimulate increased reporting of adverse drug reactions ‐ a way of dealing with an old problem? Eur J Clin Pharmacol. 2006;62(5):381–5. 16572320 10.1007/s00228-005-0072-0

[29] Feely J , Moriarty S , O'Connor P . Stimulating reporting of adverse drug reactions by using a fee. Br Med J. 1990;300(6716):22–3. 2105117 10.1136/bmj.300.6716.22 PMC1661889

[30] Barrow PL , Jadeja M , Foy M . Establishing electronic adverse drug reaction reporting in UK primary care clinical IT systems. Drug Saf. 2012;35(10):909–10.

[31] Ribeiro‐Vaz I , Santos C , da Costa‐Pereira A , Cruz‐Correia R . Promoting spontaneous adverse drug reaction reporting in hospitals using a hyperlink to the online reporting form: an ecological study in Portugal. Drug Saf. 2012;35(5):387–94. 22468615 10.2165/11597190-000000000-00000

[32] Hobbs T . Access all areas – empowering patient care using TGA initiatives. Presented at: Pharmacy Australia Congress 2014. 2021. Accessed August 24, 2021. https://www.tga.gov.au/sites/default/files/events‐presentations‐pac‐141010.pdf

[33] Davies H , Noble PJ , Pinchbeck G , Killick DR , Diesel G . Reporting adverse events and lack of efficacy. Vet Rec. 2020;187(10):407. 10.1136/vr.m4287 33188120

[34] Trifirò G , Patadia V , Schuemie MJ , Coloma PM , Gini R , Herings R , et al. EU‐ADR healthcare database network vs. spontaneous reporting system database: preliminary comparison of signal detection. Stud Health Technol Inform. 2011;166:25–30. 21685607

[35] Patadia VK , Schuemie MJ , Coloma PM , van der Lei J , Trifirò G , Herings R , et al. Can electronic health records databases complement spontaneous reporting system databases? A historical‐reconstruction of the association of rofecoxib and acute myocardial infarction. Front Pharmacol. 2018;9:594. 29928230 10.3389/fphar.2018.00594 PMC5997784

